# *Curvularia lunata,* a rare fungal peritonitis in continuous ambulatory peritoneal dialysis (CAPD); a rare case report

**Published:** 2015-12-21

**Authors:** Haritha Subramanyam, Ramprasad Elumalai, Anupma Jyoti Kindo, Soundararajan Periasamy

**Affiliations:** ^1^Department of Nephrology, Sri Ramachandra Medical College and Hospital, Sri Ramachandra University, Chennai, India; ^2^Department of Microbiology, Sri Ramachandra Medical College and Hospital, Sri Ramachandra University, Chennai, India

**Keywords:** Continuous ambulatory peritoneal diagnosis, peritonitis, *Curvularia lunata*

## Abstract

Peritonitis is an inflammation of the peritoneum that occurs in patients with end-stage renal disease (ESRD) treated by peritoneal dialysis. Fungal peritonitis is a dreaded complication of peritoneal dialysis. *Curvularia lunata* is known to cause extra renal disease like endocarditis, secondary allergic bronchopulmonary aspergillosis and endophthalmitis. This case report presents a case of continuous ambulatory peritoneal dialysis peritonitis with this disease and its management. This case is of a 45-year-old man, presented with ESRD, secondary to diabetic nephropathy. After 3 months of hemodialysis the patient was put on continuous ambulatory peritoneal dialysis (CAPD). Local Examination at catheter site showed skin excoriation and purulent discharge. Further peritoneal dialysis (PD) fluid analysis showed neutrophilic leukocytosis and diagnosis of *Curvularia lunata* PD peritonitis.

**Implication for health policy/practice/research/medical education:**

Peritonitis is an inflammation of the peritoneum that occurs in patients with end-stage renal disease (ESRD) treated by peritoneal dialysis. Fungal peritonitis is a dreaded complication of peritoneal dialysis (PD). Curvularia lunata is known to cause extra renal disease like endocarditis, secondary allergic bronchopulmonary aspergillosis and endophthalmitis. This case report presents a case of continuous ambulatory peritoneal dialysis ( CAPD) peritonitis with this disease and its management.

## Introduction

Peritonitis is an inflammation of the peritoneum that occurs in patients with end-stage renal disease (ESRD) treated by peritoneal dialysis (PD). It typically has an infectious etiology, mainly due to gram-positive bacilli or fungus (1). Bacterial infections come from contamination during PD and fungal infections may occur subsequent to antibiotic use. Peritonitis is a major complication of PD, with high rate of morbidity and mortality in patients on continuous ambulatory peritoneal dialysis (CAPD). Fungal peritonitis is dreaded complication, accounting for 2%-7% of PD related peritonitis. It should be strongly suspected after recent antibiotic treatment for bacterial peritonitis. Comparatively fungal peritonitis is associated with higher rates of hospitalization, catheter removal, transfer to hemodialysis and death. In recent reports, several other filamentous fungi, yeasts are increasingly being isolated. We report a case of CAPD peritonitis with *Curvularia lunata* and its management.

## Case presentation

45-year-old man, farmer by occupation, presented with ESRD, secondary to diabetic nephropathy. He was on hemodialysis for three months. Subsequently, initiated on CAPD, via Swan neck, Tenckhoff catheter. He was on four exchange/day with ultrafiltration of 1 liter/day and residual urine output 700 ml/day. Patient was stable for 6 months. Later, patient developed abdominal pain and PD fluid of cloudy in color. The analysis of PD fluid, showed, neutrophilic leukocytosis (WBC; 700/µl). He was empirically treated with cefazolin, and vancomycin, intraperitoneally. His symptoms persisted for 2 weeks. At this point, he was referred to Sri Ramachandra medical Centre, Chennai, India, in view of refractory peritonitis.

**Figure 1:**
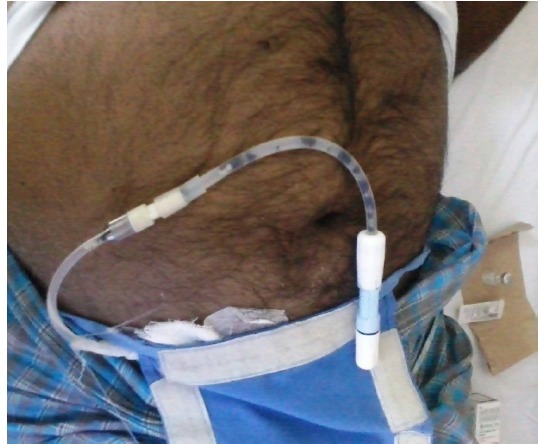
Black flakes present in CAPD catheter.

On presentation, patient was febrile and in fluid over load state. Local Examination at catheter site showed skin excoriation purulent discharge, no evidence of tunnel infection. PD catheter had black, flaky material (Figure 1). PD Fluid analysis showed neutrophilic leukocytosis. Potassium hydroxide mount*,* showed septate, fungal hyphae. Diagnosis of fungal peritonitis was made, he was started on T. Voriconazole 200 mg. PD catheter was removed. He continued to have symptoms, initiated on intravascular amphotericin B 0.6 mg/kg for 2 weeks. Fungal culture specimen showed brown conidiophores with 3-4 mm diameter septate hyphae, resulting in diagnosis of *Curvularia* *lunata* PD peritonitis. Tip of catheter grew the same organism.

## Discussion

*Curvularia* are facultative soil saprophytes, prevalent in warm and humid areas. Inhalation and skin inoculation being main routes of infection, *Curvularia* grow rapidly, forming brownish gray to black colonies. CAPD Peritonitis due to fungi *Curvularia lunata* must be suspected if black flakes are seen in the effluent of catheter (2).*Curvularia* species cause disease in humans and animals. Genus *Curvularia* comprises of several other species (3,4). Evidence from International Society for Peritoneal Dialysis (ISPD) guidelines state that species identification and minimum inhibitory concentration (MIC) values are crucial due to the emergence of resistance to azoles (5). Treatment includes of amphotericin B, voriconazole. Fluconazole prophylaxis effective in patients in preventing secondary fungal infections in patients treated for bacterial peritonitis (3). Intraperitoneal use of amphotericin causes chemical peritonitis while in use leads to poor peritoneal penetration (6). Currently our patient is doing well after catheter removal, shifted to maintenance hemodialysis (MHD). Retrospective study done in our center, during seven years period stated that *Candida albicans* and non-albicans are most prevalent (7). *Curvularia* known to cause other extra renal disease like endocarditis, secondary allergic bronchopulmonary aspergillosis and endophthalmitis. In summary, our report shows the management of CAPD associated *Curvularia lunata* peritonitis.

## Conclusion

In ESRD treated by PD, peritonitis is an inflammation with infectious etiology mainly due to gram-positive bacilli or fungus. Fungal peritonitis is associated with higher rates of hospitalization, catheter removal, transfer to hemodialysis and death. Evaluation of the patients who have* Curvularia* *lunata* peritonitis can help to rule out other etiologies and helps in the management of CAPD associated *Curvularia* *lunata* peritonitis.

## Authors’ contribution

HS, RE and SP are involved in treating the patient. AK characterized Curvularia lunata. HS, RE and SP prepared the manuscript. All authors read, revised and approved the final manuscript.

## Conflicts of interest

The authors declared no competing interests.

## Ethical considerations

Ethical issues (including plagiarism, data fabrication, double publication) have been completely observed by authors.

## Funding/Support

No funding from any source is directly associated with this study.

## References

[1] Bibashi E, Memmos D, Kokolina E, Tsakiris D, Sofianou D, Papadimitriou M (2003). Fungal peritonitis complicating peritoneal dialysis during an 11-year period: report of 46 cases. Clin Infect Dis.

[2] Varughese S, David VG, Mathews MS, Tamilarasi V (2011). A patient with amphotericin-resistant Curvularia lunata peritonitis. Perit Dial Int.

[3] Kalawat U, Reddy GS, Sandeep Y, Naveen PR, Manjusha Y, Chaudhury A (2012). Succesfully treated Curvularia lunata peritonitis in a peritoneal dialysis patient. Indian J Nephrol.

[4] Guarner J, Del Rio C, Williams P, McGowan JE Jr (1989). Fungal peritonitis caused by Curvularia lunata in a patient undergoing peritoneal dialysis. Am J Med Sci.

[5] Bhowmik D, Mahajan S, Bora M (2011). Concerns regarding the ISPD guidelines/recommendations for peritonitis due to mycobacteria. Perit Dial Int.

[6] Wong PN, Lo KY, Tong GM, Chan SF, Lo MW, Mak SK (2008). Treatment of fungal peritonitis with a combination of intravenous amphotericin B and oral flucytosine, and delayed catheter replacement in continuous ambulatory peritoneal dialysis. Perit Dial Int.

[7] Indhumathi E, Chandrasekaran V, Jagadeswaran D, Varadarajan M, Abraham G, Soundararajan P (2009). The risk factors and outcome of fungal peritonitis in continuous ambulatory peritoneal dialysis patients. Indian J Med Microbiol.

